# *In vitro* Liver Zonation of Primary Rat Hepatocytes

**DOI:** 10.3389/fbioe.2019.00017

**Published:** 2019-02-18

**Authors:** Lauren Tomlinson, Lauren Hyndman, James W. Firman, Robert Bentley, Jonathan A. Kyffin, Steven D. Webb, Sean McGinty, Parveen Sharma

**Affiliations:** ^1^MRC Centre for Drug Safety Science, Department of Clinical and Molecular Pharmacology, University of Liverpool, Liverpool, United Kingdom; ^2^Division of Biomedical Engineering, University of Glasgow, Glasgow, United Kingdom; ^3^Department of Pharmacy and Biomolecular Science, Liverpool John Moores University, Liverpool, United Kingdom; ^4^Department of Applied Mathematics, Liverpool John Moores University, Liverpool, United Kingdom; ^5^EPSRC Liverpool Centre for Mathematics in Healthcare, Department of Mathematical Sciences, University of Liverpool, Liverpool, United Kingdom

**Keywords:** liver zonation, mathematical modeling, flow system, drug-induced liver injury, *in vitro* model

## Abstract

The ability of the liver to simultaneously carry out multiple functions is dependent on the metabolic heterogeneity of hepatocytes spatially located within a liver lobule spanning from the portal triad to the central vein. This complex zonal architecture of the liver, however, makes accurate *in vitro* modeling a challenge and often standard culture systems assume a homogenous model which may lead to inaccurate translatability of results. Here, we use a combination of mathematical modeling and experimental data to demonstrate a readily constructible *in vitro* flow system capable of liver zonation in primary rat hepatocytes. We show the differential expression of zonation markers, enhanced functionality when compared to standard static cultures and zone-specific metabolism and cell damage in the presence of paracetamol, a known zone-specific toxin. This type of advanced system provides a more in-depth and essential understanding of liver physiology and pathophysiology as well as the accurate evaluation of pharmacological interventions at a zone-specific level.

## Introduction

**Figure 1:**
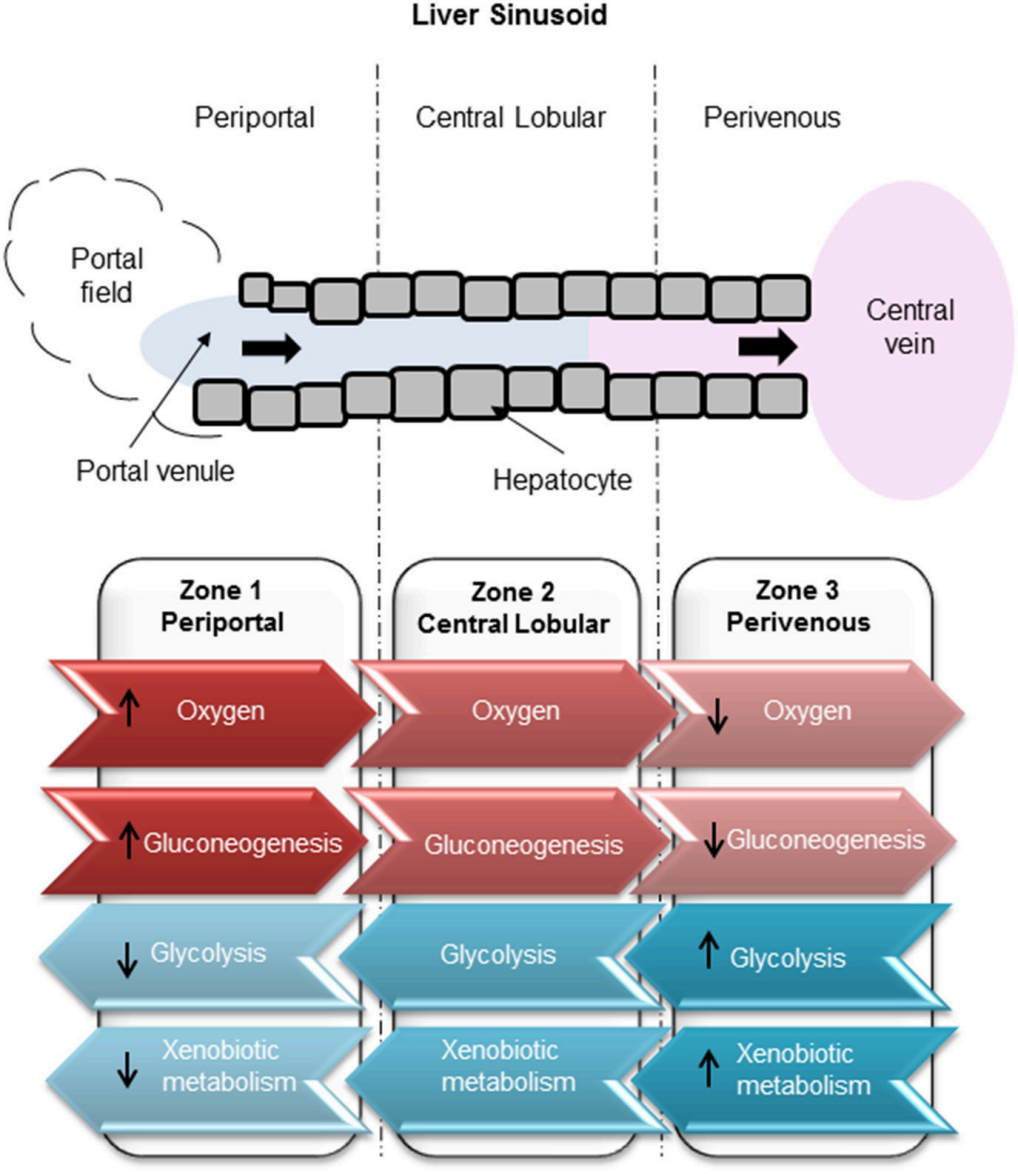
Zonation of liver metabolism. High oxygen exposure of hepatocytes in the periportal region compared to low exposure in the perivenous zone. Glucose production carried out through gluconeogenesis in the periportal zone. Glucose utilization carried out by glycolysis in the perivenous zone.

Drug-induced liver injury (DILI) represents a major global human health concern and is one of the most common side effects of many therapeutic compounds, leading to a high incidence of patient morbidity and mortality (Gaskell et al., 2016). Exposure to hepatotoxic compounds can result in liver failure, a life threatening condition usually requiring a liver transplant (Reuben et al., 2010). DILI carries a mortality rate of around 10% (Singh et al., 2016) which can be attributed to a poor understanding of the mechanisms underlying the toxic response and to a lack of appropriate tools for the prediction of toxic outcome. Current *in vitro* test systems include simple liver-derived 2 dimensional (2D) cell-based models that are poorly predictive of toxicity (Williams et al., 2013). Further complexity arises since it has previously been determined that hepatocytes in the liver are a heterogeneous population and, that in order to cope with an immense spectrum of functions which are performed simultaneously, liver cells show a considerable heterogeneity and functional plasticity known as metabolic zonation (Colnot and Perret, 2011). Hepatocytes within the liver sinusoid are exposed to varying gradients of oxygen, nutrients, hormones, and metabolites giving rise to zonation whereby cells along the sinusoid have vastly different levels of gene expression and metabolic competence (Kietzmann, 2017). The 3 main zones (Figure 1) along a sinusoidal unit, namely periportal (PP), central lobular (CL) and perivenous (PV), are functionally and biochemically different affecting key functions such as ammonia detoxification, glucose/energy metabolism (PP), and xenobiotic metabolism (PV) (Colnot and Perret, 2011). Hepatocytes located in the periportal region surround the portal triad, in close proximity to the blood, which is associated with zone 1. Perivenous hepatocytes associated with zone 3 are situated near the efferent centrilobular vein. Zone 2 consists of hepatocytes which are positioned in the midlobular region (Birchmeier, 2016; Kietzmann, 2017). Therefore, standard cell culture techniques that assume a homogeneous population may not provide the best biological test model to emulate DILI. It is well-established that an oxygen gradient exists throughout the three liver zones (Colnot and Perret, 2011; Birchmeier, 2016; Kietzmann, 2017) and that this gradient may contribute in part to the differential metabolic functions along the liver sinusoid (Allen and Bhatia, 2003). The liver receives highly oxygenated blood from the hepatic artery, whereas oxygen depleted blood is associated with the hepatic portal vein. In contrast, hepatocytes cultured under standard conditions in an *in vitro* environment receive a uniform oxygen supply thereby not accurately emulating an *in vivo* environment.

Using a combination of mathematical modeling and experimental data, we have designed and developed a zonated *in vitro* liver model using 3 chambers in the Quasi Vivo system^1^. By varying the elevation of cells within the system, the oxygen tension that the cells are exposed to also varies. The resulting model is therefore more representative of an *in vivo* system in which cells are exposed to multiple solute gradients, shear stress, circulating nutrients and mechanical compression. By using primary rat hepatocytes (PRH), we have shown that the cells exhibit differential protein expression and toxicity profiles when exposed to known hepatotoxins, mimicking a response more like that seen *in vivo*.

## Methods

### Primary Rat Hepatocyte Sandwich Culture

Hepatocytes were isolated from Wistar rats. This study was carried out in accordance with the principles of the Basel Declaration and recommendations of ARRIVE guidelines issued by the NC3Rs, with approval from the University of Liverpool's AWERB committee (Animal Welfare and Ethical Review Body). The University is a signatory on the concordat on openness in animal research and all work was authorized by the Home Office under the Animals (Scientific Procedures) act 1986 and the EU Directive. Primary rat hepatocytes were cultured using Williams' E media, supplemented with 10% heat inactivated FBS, 1% transferrin, 1% L-glutamine, 1% penicillin streptomycin, and 100 nM dexamethasone. Glass cover slips (13 mm) were collagen-coated 1:60 with rat tail collagen 1 and acetic acid prior to cell seeding. Cells were seeded at a density of 1 × 10^6^ per well in a 24-well plate. Following 3 h post seeding, cover slips were coated with 1:80 dilution of Matrigel (Corning) in Williams' E media, prior to overnight incubation at 37°C with a humidified atmosphere of 5% CO_2_.

### Kirkstall Quasi Vivo Flow System

**Figure 2:**
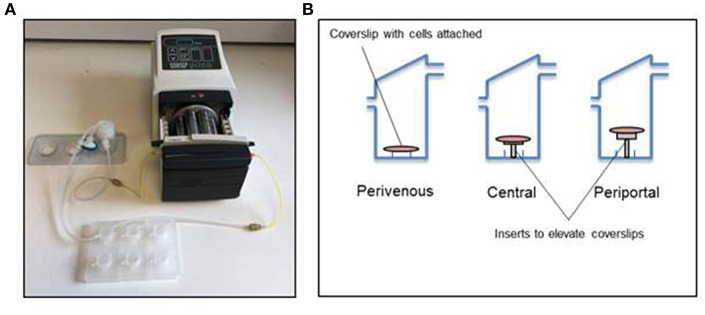
Kirkstall QV-900 setup. **(A)** Kirkstall QV-900 plates are connected using Tygon silicone tubing to reservoir bottle (Kirkstall) and then connected to a multi-channel cassette pump (Watson-Marlow) using manifold tubing (Watson-Marlow). **(B)** Schematic of setup on the inside of the QV-900 wells.

The Kirkstall QV-900 is a millifluidic media perfusion system that enables cells to be cultured in a slow-flow environment (Figure 2A). The QV-900 was washed in 20 ml of 70% IPA followed by two washes with Hanks solution. The glass cover slips containing the PRH sandwich cultures were placed in to each chamber on three different levels determined by the mathematical modeling to represent the perivenous, central, and periportal zones (Figure 2B). Each QV-900 circuit consisted of a total of 20 ml of Williams' E media flowing through the cells chamber, tubes and reservoir bottle. The system was set up at a flow rate of 150 μl/min and incubated at 37°C with a humidified atmosphere of 5% CO_2_ for 72 h. Comparisons were made to static cultures in which cells were grown in identical conditions but placed in a standard 6-well cell culture plate (Corning) without flow.

### Mathematical Modeling of Zonated Liver

We developed a mathematical model of fluid flow, oxygen transport and consumption of oxygen by the cells within the QV-900 chambers. The fluid flow was described by the steady Navier-Stokes equations, with the transport of oxygen described by a convection-diffusion equation. Consumption of oxygen by the cells was modeled via Michaelis-Menten kinetics and implemented through a flux boundary condition. For full details of the model equations, boundary conditions, solution method and parameters, the reader is referred to the Supplementary Material.

The mathematical model was employed to estimate the oxygen concentration at the cell surface for a variety of chamber depths, with the aim of generating a physiologically relevant oxygen gradient across 3 chambers, representing the periportal, central, and perivenous zones. The chamber depths were chosen such that the oxygen concentration ranged from approximately 4–15% (Mazzei et al., 2010) across the 3 chambers. In order to alter the depth at which cells were cultured in the chambers, inserts were produced with the heights determined from the mathematical modeling.

### Adenosine Triphosphate (ATP) Cell Viability Assay

ATP cell viability assay was performed using CellTiter-Glo Luminescent Cell Viability Assay (Promega) following manufacturer's instructions.

### Bradford Assay

Total protein concentration was calculated using Bradford Reagent (Sigma) using manufacturer's instructions. Briefly, a standard curve was generated using known concentrations of bovine serum albumin (Sigma) and used to calculate sample concentrations using a Varioskan Flash plate reader (Thermo Scientific).

### Lactate Dehydrogenase (LDH) Release

LDH release was measured using Cytotoxicity detection kit (Sigma) following manufacturer's instructions. Briefly, total LDH was calculated by the summation of the LDH measured in the cell lysate and media and LDH released then calculated in the media as a proportion of the total.

### Albumin Secretion

Media from static and flow conditions were centrifuged at 3,000 g for 10 min, with the supernatant collected for analysis. A series of albumin standards were prepared, alongside the media samples and each subsequent step was carried out at room temperature. A total volume of 50 μL for each standard and sample was placed in to a 96-well plate for 1 h. Standards and samples were washed five times in 1 × wash buffer. A total volume of 50 μL of 1 × Biotinylated Albumin antibody was added to each well for 1 h, prior to a series of washes as previously described. A total volume of 50 μL of 1 × SP Conjugate (100 × Streptavidin-Peroxidase Conjugate diluted 1:100 with 1 × Diluent N) was added to each well for 30 min, prior to the wash step. A total volume of 50 μL of Chromogen Substrate was added to each well for 10 min. In order to inhibit the reaction, a final volume of 50 μL of Stop solution was added to each well. The absorbance was recorded at 450 nm on a microplate reader.

### Western Blot

Samples were suspended in lysis buffer (250 mM Sucrose, 50 mM Tris pH 7.4, 1 MgCl, and 0.2 % (v/v) Triton X-100). The cell lysates were then centrifuged at 5,000 g at 4°C for 15 min. A total of 20 μg of protein was analyzed using standard immunoblotting analysis. Membranes were blocked overnight in 5% milk in blocking buffer (0.2% Triton X-100 in PBS) at 4°C. The primary antibodies used were; Glutamine synthetase (GS, Abcam) 1:1,000, CYP3A4 (Abcam) 1:1,000, carbamoyl-phosphate synthase 1 (CPS1, Abcam) 1:1,000, Arginase 1 (Arg1, Abcam) 1:1,000, β-actin (Abcam) 1:1,000.

### Zone Specific Toxicity

Cells were incubated under flow for 24 h, after which they were dosed with 50 mM paracetamol, a zone specific toxin, dissolved in the circulating Williams' E media for 48 h.

## Results

### Mathematical Modeling of an *in vitro* Liver

Firstly, simulations were performed assuming that the cells were cultured at the base of the chamber to obtain a baseline for the cell surface oxygen concentration. The minimum cell surface oxygen concentration for this configuration was found to be approximately 4% (see Supplementary Material), whereas the mean value was approximately 6% (Table 1). Therefore, cells cultured at the base of the chamber were assumed to be representative of the perivenous zone. The height at which the cells were assumed to be cultured was subsequently raised by increments of 1 mm (see Supplementary Material) until a mean cell surface oxygen concentration of approximately 15% was obtained. This corresponded to raising the cells by 7 mm and so this height was selected for the chamber representing the periportal zone. Finally, an intermediate height of 4 mm was selected for the chamber representing the central zone. Therefore, inserts with heights 4 mm and 7 mm were created for use in the experiments. The results of all preliminary simulations are provided in the Supplementary Material.

**Table 1:** Mean oxygen concentration and magnitude of shear stress at the cell surface for each liver zone, periportal (cells raised by 7 mm), central (cells raised by 4 mm), and perivenous (cells raised by 0 mm i.e., cultured at the base of the chamber).

**Liver zone**	**Mean oxygen concentration at the cell surface (mol/m^**3**^) (O_**2**_%)**	**Mean magnitude of shear stress at cell surface (Pa)**
Periportal	0.15 (15%)	1.69 × 10^−6^
Central	0.12 (12%)	4.32 × 10^−7^
Perivenous	0.06 (6%)	2.50 × 10^−8^

**Figure 3:**
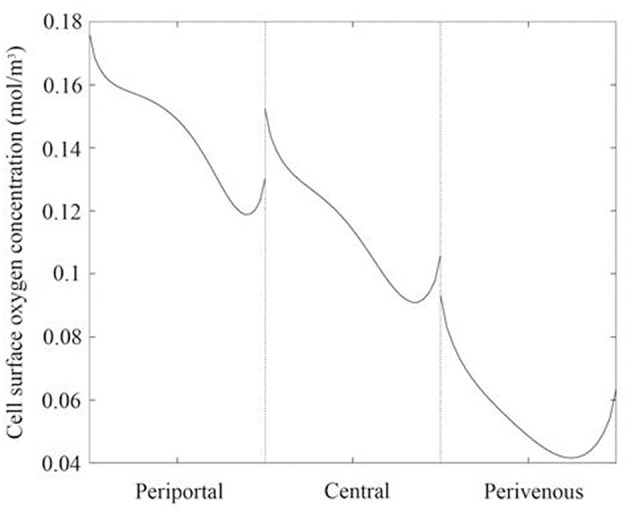
Oxygen concentration profile across the center of the cell surface for each liver zone. Periportal (cells raised by 7 mm), central (cells raised by 4 mm), and perivenous (cells raised by 0 mm i.e., cultured at the base of the chamber).

Whilst raising the cells within the chambers enables exposure to higher oxygen concentrations, a potential drawback is that the fluid environment also varies with height, leading to higher cell surface shear stress which has the potential to damage the cells. We therefore also calculated the variation in shear stress with height. For each liver zone, Table 1 displays the simulated mean oxygen concentration and magnitude of shear stress at the cell surface and Figure 3 shows the oxygen concentration profile across the center of the cell surface.

### Verification of Zonation

**Figure 4:**
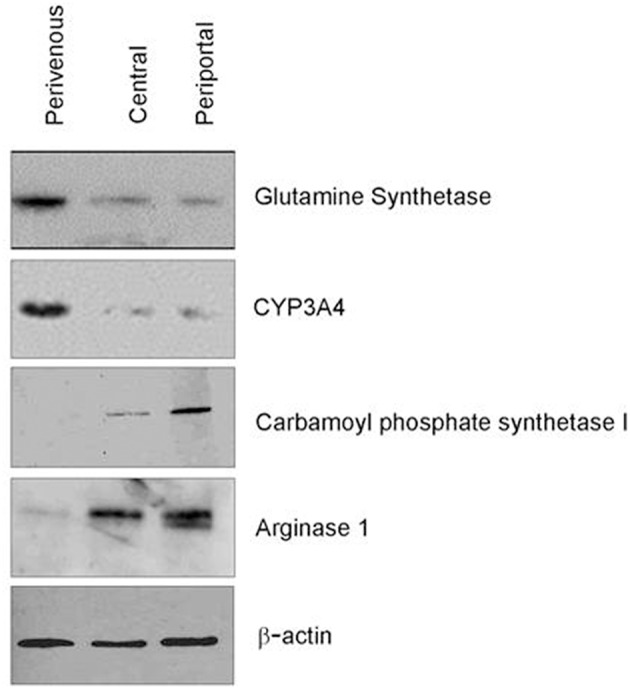
Verification of zonation. Western blot analysis of zone specific protein markers.

Confirmation of zonation by immunoblotting showed differential expression of specific zonation markers in each chamber (Figure 4). Glutamine synthetase (GS) converts ammonia into glutamine in the perivenous hepatocytes and drug detoxification enzymes CYP 3A4 were shown to be expressed highest in the perivenous chamber whereas carbamoyl phosphate synthetase 1 (CPS1) and arginase 1 (Arg1), two enzymes within the urea cycle, were found predominantly in the periportal zone.

**Figure 5:**
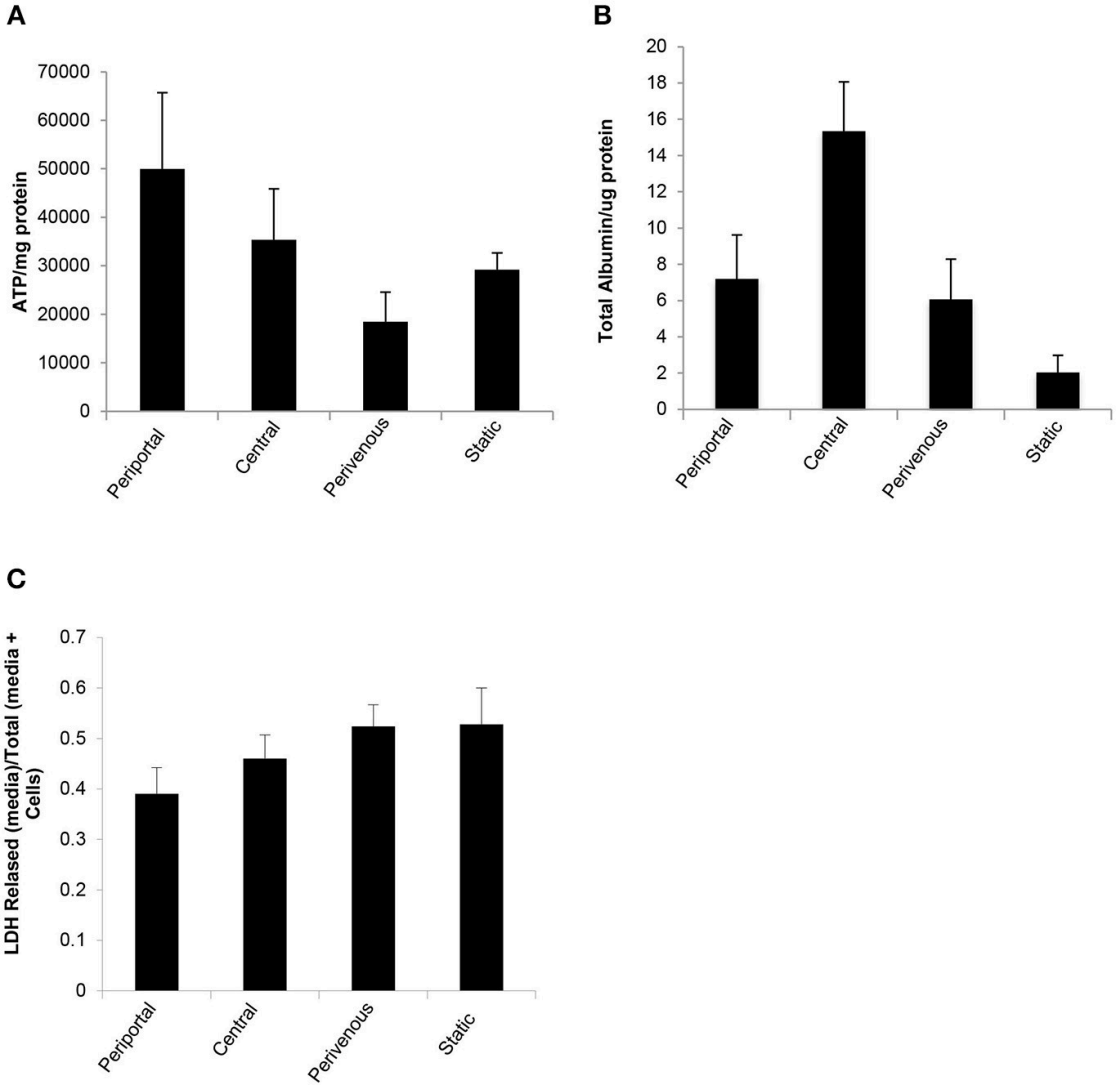
Functional analysis of PRH in each zone compared to standard 2D static conditions. After 48 h under flow conditions or static conditions functional parameters were assessed **(A)** ATP (*n* = 4), **(B)** Albumin secretion (*n* = 4). Both were normalized to protein concentration calculated using a Bradford assay, and **(C)** Cytotoxicity of shear stress analyzed by LDH assay. Data shown as LDH released (in media)/Total LDH (present in cells + media).

The *in vitro* system was maintained in full nutritional state and in keeping with the fact that glycolysis is accelerated in a fed state and gluconeogenesis is diminished (Phillips et al., 2002) we found a significantly greater release of ATP in the periportal zone, where gluconeogenesis is predominate (Figure 1) when compared to the perivenous zone (*p* < 0.05, students *t*-test) (Figure 5A). The low shear stress felt by the cells ensures that cells are not lost in the flow system and retain functionality as evident by ATP levels (Figure 5A) however, to account for any loss values were normalized to protein content carried out using a standard Bradford assay (see methods). As a measure of cell health we next analyzed albumin production and found a 3 fold increase in the perivenous region, 3.5 fold increase in the periportal region and a 7.5 fold increase in the central region when compared to cells grown under standard static conditions (Figure 5B). In order to ascertain if the application of shear stress damaged the cells we next determined levels of lactate dehydrogenase (LDH) as an established biomarker of liver damage. Figure 5C shows that no additional LDH was released in the cells under the flow systems when compared to those grown under standard static conditions.

### Zone Specific Metabolism

**Figure 6:**
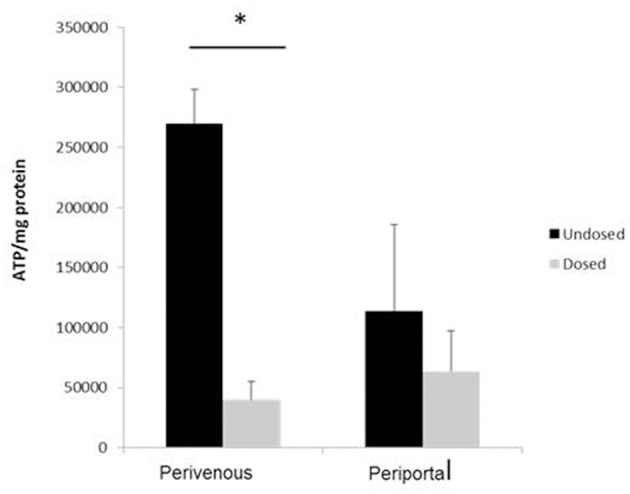
Confirmation of zone specific metabolism. Cells were dosed with 50 mM paracetamol for 48 h. Viability (calculated using ATP assay) was normalized to protein concentration calculated using a Bradford assay. *n* = 3, student's test used for statistical analysis. ^*^p < 0.05.

Paracetamol is metabolized in the liver predominantly by CYP enzymes where it is metabolized to the cytotoxic intermediate NAPQI, which in turn depletes glutathione, leading to cell death after an overdose (McCarty et al., 2016). We next incubated the cells with paracetamol for 48 h and showed that significantly greater cell death occurred at the perivenous zone when compared to the periportal confirming that our system has zone specific drug metabolism (Figure 6).

## Discussion

Accurate *in vitro* modeling is essential to data translatability *in vivo*. Despite the distinct metabolic zonation signature of the *in vivo* liver, many standard 2D and 3D culture systems overlook the metabolic heterogeneity of the 3 distinct zones largely due to the complexity of the generation of an *in vitro* zonated model (Dash et al., 2009). To facilitate this process and to make a zonated *in vitro* liver model more widely available, we have modeled fluid flow and oxygen transport within the chambers of a Quasi-vivo 900 system^1^. We chose the QV-900 for two main reasons. Firstly, it allows one to expose cells to flow (as we would have along the hepatic sinusoid) at relatively low shear stress levels which assures the conservation of cell numbers in our system. Secondly, it is straightforward to customize the cell culture depth within the chambers. Our experimental and mathematical modeling efforts allow for the simulation of flow patterns, shear stress and physiological oxygen levels similarly experienced by the *in vivo* cells within the distinct zones of the liver sinusoid. We demonstrate differential expression of established zone specific markers and metabolic cell health functions such as ATP and albumin production that supersede those seen in standard 2D culture systems. To further evaluate cell death or damage we used the LDH analysis which is a standard method of evaluating dead or dying cells (Smith et al., 2011; Zahedifard et al., 2015; Kumar et al., 2018). As we show in Figure 5C there is no additional LDH release in any of the zones when compared to the static cells indicating that the low shear stress applied in this system does not damage the cells.

A major concern in using primary hepatocytes for drug safety analysis is their rapid dedifferentiation in static culture resulting in a loss of hepatic phenotype and functionality within hours of the isolation procedure (Yixin et al., 2012; Lauschke et al., 2016; Lauschkek et al., 2016). However, research has suggested that the addition of fluid flow maintains the expression of genes involved in xenobiotic and drug metabolism and transport over a sustained culture period (Vinci et al., 2011; Lauschkek et al., 2016). Here we show a zonated drug toxicity response after exposure to paracetamol 48 h after initial isolation of cells. Paracetamol toxicity was seen predominantly in the perivenous zone where hepatocytes are known to have a high zonal expression of the CYP450 enzymes responsible for the generation of the toxic metabolite NAPQI (Henderson et al., 2000; Mazer and Perrone, 2008).

Research into factors driving liver zonation have reported several mechanisms by which the different metabolic zones arise (Kietzmann, 2017). These include gradients of morphogens such as Wnt, hedgehog, hormones or growth factors as well as oxygen (Colnot and Perret, 2011; Kietzmann, 2017). Although it was previously deemed insufficient for oxygen alone to drive zonation (McCarty et al., 2016), more recent thinking has implicated that oxygen level variations may drive Wnt/β-catenin pathway which is considered to be a master driver of hepatic zonation (Kietzmann, 2017). The low oxygen content in the perivenous zone has been implicated to activate β-catenin which acts as a positive regulator of perivenous genes including glutamine synthetase (GS) and in turn negatively regulates arginase 1 (Arg1). Within the perivenous zone of our system we observe an upregulation of GS, an essential enzyme in nitrogen metabolism, and down regulation of Arg1, a key enzyme in the urea cycle supporting the concept of an oxygen-controlled element in the mechanism of the Wnt/β-catenin pathway.

We would like to emphasize that a number of assumptions have been made in our mathematical model (see Supplementary Material). In particular, in common with mathematical modeling of most biological systems, it is a challenge to obtain a reliable and consistent set of parameter values. In this work, we utilized Michaelis-Menten parameter values reported in the literature (Mazzei et al., 2010) in a study which conducted computational modeling of a bioreactor system similar to the one modeled here. Although variations in these parameters will have an influence on the magnitude of oxygen gradient simulated, the trends should be the same. Our goal was not to accurately *predict* concentration gradients. Rather, the focus of our work was to demonstrate the feasibility of creating oxygen gradients that result in differential expression of zonation markers and enhanced functionality of hepatocytes, and we have achieved this in the current study. A useful next step would be to obtain accurate measurement of the Michaelis-Menten parameters associated with hepatocytes and to validate the resulting oxygen gradients predicted in our model. This would enable us to confirm the predictive nature of the model and in turn would allow us to assess the effect of varying future experimental conditions.

Over the past decade, several organ-on-a-chip systems have been developed (Huh et al., 2013; David et al., 2017) with microfluidic flow systems in order to emulate a more *in vivo* like system. However, several limitations of these systems remain, including complex operational control, custom (non-commercial) chip design (Halldorsson et al., 2015; Kang et al., 2018) the presences of bubbles that may damage cells (Sbrana and Ahluwalia, 2012; Bhatia and Ingber, 2014), handling of small volume and ultimately having a limited sample size for downstream applications. However, our zonated system described here takes advantage of a commercially available system specifically designed to remove air bubbles (Sbrana and Ahluwalia, 2012) that allows the seeding and recovery of 1 × 10^6^ zone-specific cells for all manner of downstream applications and its millifluidic nature further reduces technical complications. We provide here a user friendly operational system optimized to emulate a zonated liver that will allow a more *in vivo* like assessment of liver physiology and pathophysiology as well as evaluation of drug metabolism and safety.

## Author Contributions

LT, JWF, RB, JAK, and PS carried out the molecular lab work and participated in data analysis. LH, SM, and SDW carried out mathematical modeling. PS, SM, and SDW conceived and designed the study and helped draft the manuscript. All authors gave final approval for publication.

### Conflict of Interest Statement

The authors declare that the research was conducted in the absence of any commercial or financial relationships that could be construed as a potential conflict of interest.

## Footnotes

^1^Available online at: http://www.kirkstall.com/about-kirkstall/
