# Supplementary material for: In vitro Liver Zonation of Primary Rat Hepatocytes
Source: Front Bioeng Biotechnol. 2019 Feb 18;7:17. doi: 10.3389/fbioe.2019.00017 (PMC6387900; doi:10.3389/fbioe.2019.00017)
Supplement: Supplementary file 1 [file Data_Sheet_1.pdf]

Supplementary material for “*In vitro* liver zonation of primary rat hepatocytes”

Computational geometry

A 3D representation of a QV-900 chamber was constructed in COMSOL Multiphysics (Figure 1). The overall height of the chamber ranges from 18.6 - 20.7 mm at the inlet and outlet sides, respectively, and the diameter of the chamber is taken to be 16 mm, although it is noted that this varies marginally with chamber height. The inner diameter of the inlet is 1 mm whilst the outlet has a larger inner diameter of 1.8 mm. Initially, the cells are assumed to be cultured on a 13 mm cover slip at the base of the chamber and where the cells are raised in 1 mm increments, we simply amend the geometry shown in Figure 1 by adjusting the overall height of the chamber.

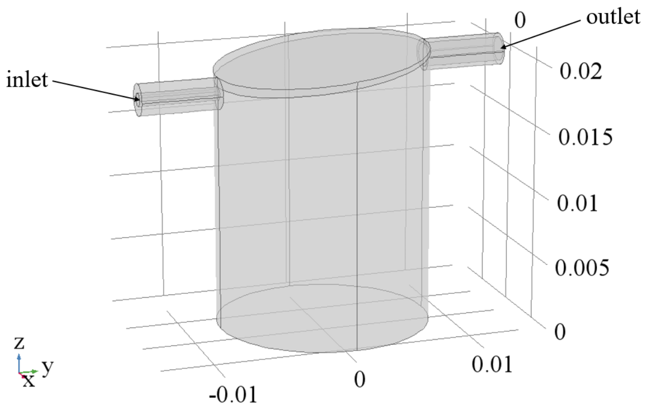

Figure 1: Idealised 3D geometry of a QV-900 chamber, showing the orientation of the x, y and z axes. The origin is chosen to be located at the centre of the base.

Model equations

Assuming the fluid is incompressible and Newtonian, the flow velocity and pressure are described using the continuity and Navier-Stokes equations:

$$\nabla \cdot \mathbf{u} = 0, \tag{1}$$

$$\rho \frac{\partial \mathbf{u}}{\partial t} + \rho(\mathbf{u} \cdot \nabla)\mathbf{u} = -\nabla p + \mu \nabla^2 \mathbf{u}, \tag{2}$$

where $\mathbf{u}$ ($\mathrm{m\ s^{-1}}$) is the velocity field, p (Pa) is the pressure, ρ is the fluid density and μ is the dynamic viscosity. Whilst it is recognised that the parameters ρ and μ may change according to temperature and the specific fluid used, here the values are chosen to represent water at 37°C. The transport of oxygen through the fluid is described using a convection-diffusion equation:

$$\frac{\partial c}{\partial t} + (\mathbf{u} \cdot \nabla)c \quad = \quad D\nabla^2 c, \tag{3}$$

where c ($\mathrm{mol\ m^{-3}}$) is the concentration of oxygen and D is the diffusion coefficient of oxygen in water. Finally, the consumption of oxygen by the cells is described by Michaelis-Menten kinetics and is represented by the following flux boundary condition at the base of the chamber:

$$\mathbf{n} \cdot (-D\nabla c + \mathbf{u}c) \quad = \quad d\frac{V_{max}c}{K_m + c}, \tag{4}$$

where d is the cell density, V_{max} is the maximum oxygen consumption rate, K_m is the Michaelis-Menten constant and $\mathbf{n}$ is an outward facing normal.

Initial and boundary conditions

Initially, the fluid velocity and oxygen concentration are zero ($\mathbf{u} = \mathbf{0}$, $c = 0$) in the chamber. A parabolic velocity profile (derived from the input flow rate) and a constant supply of oxygen ($c = c_{in}$) are assumed at the inlet. At the outlet, zero pressure ($p = 0$) and a convective flux ($-\mathbf{n} \cdot D\nabla c = 0$) are assumed. No slip and no penetration conditions ($\mathbf{u} = \mathbf{0}$) are imposed on all walls of the chamber, and it is assumed that the chamber walls are impermeable to oxygen so a zero flux condition of the form $\mathbf{n} \cdot (-D\nabla c + \mathbf{u}c) = 0$ is also imposed.

Parameter values

Table 1 displays the parameter values that were used in the simulations. Note we have assumed that the cells possess the same maximum oxygen consumption rate (V_{max}) and Michaelis-Menten constant (K_m) regardless of the specific zone they reside in. This does *not* imply a fixed

oxygen consumption rate across the three zones: since the cells are exposed to lower oxygen concentrations due to their lower elevation, the consumption rate decreases as we move from the periportal zone to the perivenous zone.

Parameter description	Value	Reference
Fluid density (ρ)	9.94×10^2 kg m^{-3}	[1]
Fluid dynamic viscosity (μ)	6.89×10^{-4} Pa s	[1]
Oxygen diffusion coefficient in water (D)	3.00×10^{-9} m^2 s^{-1}	[2]
Inlet oxygen concentration (c_{in})	0.21 mol m^{-3}	[2]
Maximum oxygen consumption rate (V_{max})	4.80×10^{-17} mol cell^{-1} s^{-1}	[2]
Michaelis-Menten constant for oxygen consumption (K_m)	6.60×10^{-4} mol m^{-3}	[2]
Cell number (n)	2.00×10^5 cell	$*$
Area covered by the cells on 13mm cover slip (A)	1.33×10^{-4} m^2	this study
Cell density ($d = n/A$)	1.50×10^9 cell m^{-2}	this study
Input flow rate (Q)	150 μL min^{-1}	this study

Table 1: Parameter values. *1 million cells are used in the experiments but it is assumed that only 20% of the cells attach.

Solution method

COMSOL Multiphysics, a commercially available finite element analysis software, was used to perform the simulations in this study. The 'Laminar Flow' and 'Transport of Diluted Species' packages were used to model the fluid flow and oxygen transport, respectively. The built-in physics-controlled 'Finer' mesh setting was utilised since reducing to the 'Extremely fine' setting altered the results by less than 1%. For the purposes of this study, the steady version of the above equations were solved since steady-state is assumed to be achieved rapidly in the experiments.

Results

Figure 2 illustrates the oxygen concentration profile across the centre of the cell surface when the cells are raised in 1 mm increments from the base of the chamber to 7 mm above the base of the chamber, and Table 2 displays the simulated minimum, mean and maximum cell surface oxygen concentration and magnitude of shear stress for all considered cell depths.

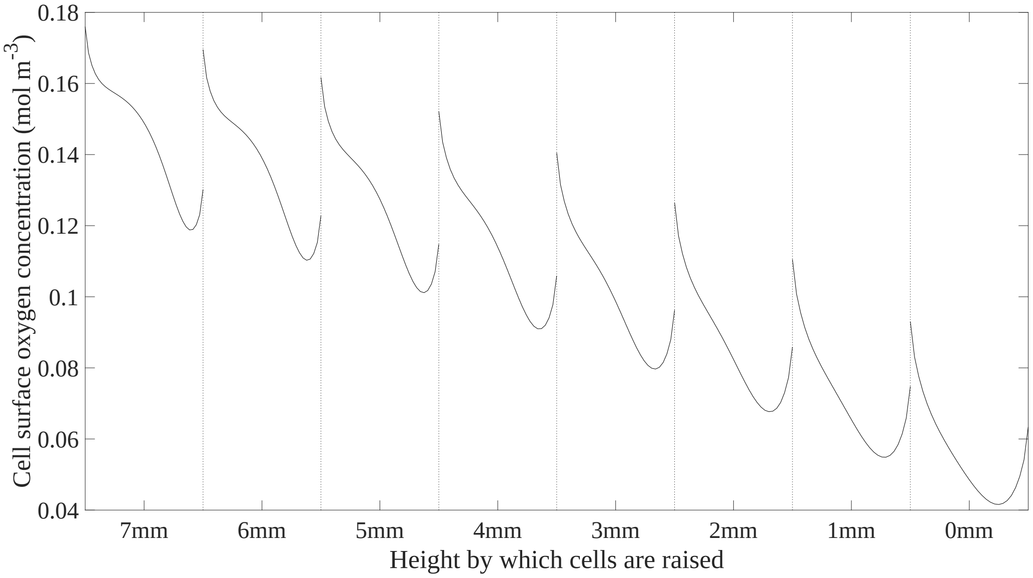

Figure 2: Oxygen concentration profile across the centre of the cell surface for various cell depths.

Height by which cells are raised (mm)	Minimum cell surface oxygen $(\mathrm{mol\ m^{-3}})$	Mean cell surface oxygen $(\mathrm{mol\ m^{-3}})$	Maximum cell surface oxygen $(\mathrm{mol\ m^{-3}})$
7	0.12	0.15	0.18
6	0.11	0.14	0.17
5	0.10	0.13	0.16
4	0.09	0.12	0.15
3	0.08	0.10	0.14
2	0.07	0.09	0.13
1	0.05	0.07	0.11
0	0.04	0.06	0.09

Height by which cells are raised (mm)	Minimum cell surface shear stress (Pa)	Mean cell surface shear stress (Pa)	Maximum cell surface shear stress (Pa)
7	2.56×10^{-8}	1.69×10^{-6}	3.50×10^{-6}
6	1.98×10^{-8}	1.10×10^{-6}	2.38×10^{-6}
5	1.30×10^{-8}	7.00×10^{-7}	1.57×10^{-6}
4	8.79×10^{-9}	4.32×10^{-7}	1.01×10^{-6}
3	5.74×10^{-9}	2.54×10^{-7}	6.23×10^{-7}
2	3.74×10^{-9}	1.37×10^{-7}	3.63×10^{-7}
1	3.00×10^{-9}	6.46×10^{-8}	1.96×10^{-7}
0	2.03×10^{-10}	2.50×10^{-8}	9.02×10^{-8}

Table 2: Minimum, mean and maximum oxygen concentration and magnitude of shear stress at the cell surface for a variety of cell depths.

References

[1] J. C. Crittenden, R. R. Trussell, D. W. Hand, K. J. Howe, and G. Tchobanoglous. *MWH's Water Treatment: Principles and Design, Third Edition*. John Wiley & Sons, 2012.

[2] D. Mazzei, M.A. Guzzardi, S. Giusti, and A. Ahluwalia. A low shear stress modular bioreactor for connected cell culture under high flow rates. *Biotechnology and Bioengineering*, 106:127–137, 2010.
